# Attitudes towards the implementation of universal umbilical artery lactate analysis in a South African district hospital

**DOI:** 10.1186/s12884-016-0968-y

**Published:** 2016-07-18

**Authors:** Emma R. Allanson, Kate Grobicki, Robert C. Pattinson, Jan E. Dickinson

**Affiliations:** School of Women’s and Infants’ Health, Faculty of Medicine, Dentistry and Health Sciences, University of Western Australia, Crawley, Australia; SAMRC Maternal and Infant Health Care Strategies unit, Department of Obstetrics and Gynaecology, University of Pretoria, Pretoria, South Africa; Zithulele Hospital, Eastern Cape, South Africa

**Keywords:** Umbilical artery lactate, Implementation, Attitudes, Training, South Africa

## Abstract

**Background:**

Of the 5.54 million stillbirths and neonatal deaths occurring globally each year, a significant amount of these occur in the setting of inadequate intrapartum care. The introduction of universal umbilical artery lactate (UA) measurements in this setting may improve outcomes by providing an objective measurement of quality of care and stimulating case reflection, audit, and practice change. It is important that consideration is given to the barriers and facilitators to implementing this tool outside of a research setting.

**Methods:**

During the period 16/11/2014 -13/01/2015, we conducted a training course in cardiotocograph (CTG) interpretation, fetal physiology, and the sampling and analysing of UA lactate, with a pre and post questionnaire aimed at assessing the barriers and facilitators to the introduction of universal UA lactate in a district hospital in the Eastern Cape, South Africa.

**Results:**

Thirty-five pre-training questionnaires available (overall response rate 95 %) and 22 post training questionnaires (response rate 63 %) were available for analysis. Prior to training, the majority gave positive responses (strongly agree or agree) that measuring UA lactate assists neonatal care, is protective for staff medicolegally, and improves opportunities for audit and teaching of maternity practice (*n* = 33, 30, 32; 94.4 %, 85.7 %, 91.4 % respectively). Respondents remained positive about the benefits post training. An increased workload on medical or midwifery staff was less likely to be seen as barrier following training (71 vs. 38.9 % positive response, *p* = 0.038). A higher rate of respondents felt that expense and lack of equipment were likely to be barriers after completing training, although this wasn’t significant. There was a trend towards lack of time and expertise being less likely to be seen as barriers post training.

**Conclusion:**

The majority of participants providing intrapartum care in this setting are positive about the role of universal UA lactate analysis and the potential benefits it provides. Training aids in overcoming some of the perceived barriers to implementation of universal UA lactate analysis.

## Background

Umbilical cord blood gas analysis immediately post-delivery is common practice in many obstetric units [1], as a tool for predicting adverse neonatal outcome to facilitate earlier intervention and as an objective defence against an intrapartum hypoxic insult. Additionally, objective feedback to health care providers on the fetal response to intrapartum events may stimulate case reflection, quality of care audit, and change in practice (with potentially altered clinical outcomes) [2]. While paired umbilical artery and vein samples that are analysed for pH, PO2, PCO2 and base excess have traditionally been the gold standard, umbilical artery (UA) lactate can also provide a technically simple and sensitive measure of fetal hypoxia to aid in the prediction of neonatal morbidity and mortality [3–6].

There are few studies (all of which are small select cohort or case control studies) outside of high income settings exploring the role of UA lactate in predicting acidosis and/or clinical outcomes [7–14]. Low resource settings account for most of the 5.54 million stillbirths and neonatal deaths occurring globally each year [15, 16]. There are an estimated 1.19 million intrapartum stillbirths [17] and more than 200,000 neonatal deaths due to intrapartum hypoxia related encephalopathy, contributed to significantly by inadequate intrapartum care [18]. The introduction of universal UA lactate measurement in these resource poor settings may contribute to reductions in poor outcomes as result of improved recognition of intrapartum hypoxia by providing an objective measurement of quality of care and stimulating case reflection, audit, and practice change.

Given the potential benefits of universal UA lactate assessment at birth, and in view of giving consideration to how such an intervention would be implemented outside of a research environment in a low resource setting, we conducted a study to assess the attitudes and barriers to the implementation of universal UA lactate in a small district hospital in South Africa. This was a secondary research study developed as an offshoot of a larger trial of universal UA lactate in a large university hospital, in Pretoria, South Africa.

## Methods

Zithulele Hospital is a district hospital in the Eastern Cape of South Africa that conducted 1923 deliveries in 2013/2014, with a perinatal mortality rate for all babies greater than 1000gr of 24.6 per 1000 [19].

During the period 16/11/2014 -13/01/2015, we conducted a training course in CTG interpretation, fetal physiology, and the sampling and analysing of UA lactate, with a pre and post questionnaire aimed at assessing the barriers and facilitators to the introduction of universal UA lactate. At the time of the study, there was no labour ward cord blood gas analyser, and cord blood case analysis was not routinely done following delivery. All medical, midwifery and nursing staff providing maternity care at Zithulele hospital were invited to participate in the study.

After providing consent, staff completed a baseline questionnaire, consisting of demographic information and 20 statements with options for answers of strongly agree, agree, neutral, disagree and strongly disagree. The statements were structured around: attitudes towards perinatal audit, CTG monitoring and cord lactate blood sampling; confidence in the attainment of cord lactate blood; confidence in the potential benefits of knowing umbilical artery lactate levels; and barriers to implementation of CTG/lactate training and universal measurement of cord lactate values. This was adapted from White CR et al. [20] with permission. A CTG and fetal physiology training program (adapted from the King Edward Memorial Hospital (Perth, Western Australia) Advanced Fetal Assessment course resources with permission) consisting of a combined lecture series (physiology of fetal heart patterns, fetal acid base status, and CTG pattern recognition), and a teaching session on the steps to sample the umbilical artery and test the lactate using the Accutrend Plus™ lactate meter (Roche, Basel, Switzerland) was then conducted. Hand held lactate meters have been shown in multiple studies to correlate with laboratory based analyses [21–26]. Samples of umbilical cord were used for training, after written consent was obtained from recently delivered patients. Participants then completed a post-training questionnaire, consisting of 17 statements with the same options for answers, and structured around the same question themes as the pre-training questionnaire.

All data were analysed using SPSS Version 22.0.0.0 (IBM Corp., Armonk, USA). Descriptive statistics were presented as frequency distributions. The relationship between the 22 individual Likert items pre and post training and with various dependents (age, position, gender, and length of time providing intrapartum care) were analysed with univariate analysis using Fisher’s exact test. All tests were two tailed and *p*-values <0.05 were considered significant. The role of gender, age, position, and length of time practicing on the identification of individual barriers to the implementation of the intervention pre- and post-training were explored using ordinal regression models.

## Results

There were 35 pre-training questionnaires available for analysis; all 13 doctors providing maternity care responded, and 22 of 24 midwives or nurses providing maternal care in the facility responded (overall response rate 95 %). All participants completed one or both aspects of training (CTG interpretation/lactate physiology and UA sampling and lactate analysis). The characteristics of respondents are presented in Table 1. Three of the 22 (13.7 %) midwife/nurse respondents were male, whereas 7 (53.8 %) of the doctors were male. The majority of respondents had been providing intrapartum care for less than 5 years, were younger than 40, and had had no formal CTG training. Prior to training, the majority of respondents felt that they were confidently (agree or strongly agree) able to identify a normal (*n* = 28/35) and an abnormal (*n* = 26/35) CTG. Only 2 respondents had ever taken a UA sample, with 19 of the 28 respondents (68 %) to the Likert item “I feel confident to take blood from the umbilical artery” giving answers ranging from neutral to strongly disagree. Twenty two participants completed the post training questionnaire (response rate 63 %).

**Table 1:** Characteristics of respondents

Characteristic	*n* (%)
Position	
Midwife/nurse	22 (62.9)
Doctor	13 (37.1)
Gender	
Female	25 (71.4)
Male	10 (28.6)
Age (years)	
20–29	12 (34.3)
30–39	12 (34.3)
40–49	5 (14.3)
≥ 50	6 (17.1)
Length of time practising (years)	
< 1	14 (42.9)
1–5	9 (25.7)
6–10	5 (14.3)
> 10	3 (8.6)
Missing	3 (8.6)
Previous CTG experience	
None	5 (14.3)
Informal workplace training	17 (48.6)
Component of degree	11 (31.4)
Specific training course	2 (5.7)

Of those that responded to the Likert items grouped under ‘attitudes towards perinatal audit’, the majority agreed with the statements “I regularly attend monthly mortality meetings” (*n* = 28, 87.5 %), “perinatal mortality meetings reduce adverse outcomes” (*n* = 28, 93.3 %), “I learn new things in perinatal mortality meetings” (*n* = 28, 93.3 %), “I change my practice after perinatal mortality meetings” (*n* = 27, 90 %), and “I feel more could be done to reduce bad outcomes due to birth asphyxia” (*n* = 29, 96.7 %).

Participants were invited to provide free form comments at the end of the pre-training questionnaire. Of the 16 respondents, 11 were positive about the potential for UA lactate analysis to give feedback for “self-reflection”, to “give us as a midwife to improve the way of conducting a delivery [sic]”, and “to see its contribution toward alleviating levels of mortality rates”. One medical staff member raised concerns about the cost effectiveness of implementing UA sampling and analysis, and two medical staff members highlighted the need “to get midwives on board”.

Participants were asked pre and post training about the specific benefits of UA lactate analysis. Prior to training, the majority gave positive responses (strongly agree or agree) that an objective measurement assists neonatal care, is protective for staff medicolegally, and improves opportunities for audit and teaching of maternity practice (*n* = 33, 30, 32; 94.4 %, 85.7 %, 91.4 % respectively). Most respondents responded positively to UA lactate being cost effective (*n* = 22, 62.9 %), however 11 (31.4 %) were neutral on this issue. There was a trend towards doctors being more likely to be neutral on this issue (53.8 % of doctors compared with 20 % of midwives/nurses answered neutrally rather than having a positive response, *p* = 0.065). Respondents remained positive about the benefits of UA lactate analysis post training, with a higher rate being positive about the cost-effectiveness (*n* = 15 (75 %) gave a positive response, with 5 (21.7 %) being neutral or disagreeing with the statement).

The positive responses (agree or strongly agree) to perceived barriers pre and post training are presented in Table 2. An increased workload on medical or midwifery staff was less likely to be seen as barrier following training (71 vs. 38.9 % positive response, *p* = 0.038). A higher rate of respondents felt that expense and lack of equipment were likely to be barriers after completing the training, although this wasn’t significant. There was a trend towards lack of time and expertise being less likely to be seen as barriers post training. Age (20–39 or ≥ 40) influenced attitude to two potential barriers; respondents aged 40 or over were more likely to see encroachment of technology into the birth process (positive response 7(77.8 %) vs. 4(25 %), negative response (2) 22.2 % vs. (12) 75 %, *p* = 0.017) and decreased patient contact time (positive response 8 (80 %) vs. 2 (11.1 %), negative response 2 (20 %) vs. 16 (88.9 %), *p* = 0.001) as barriers to the implementation of UA analysis. The influence of clinical position on perception of barriers is presented in Table 3. Both prior to and after training, midwives were more likely than doctors to answer positively to encroachment of technology into the birth process and decreased patient contact time being potential barriers. Following training, midwives identified insufficient time following delivery and lack of necessary expertise as barriers far more than doctors did.

**Table 2:** Perceived barriers to introduction of universal umbilical artery lactate analysis pre and post training

Barrier	Positive response pre training *n* (%)	Positive response post training *n* (%)	*p*-value^a^
Expense	15 (78.9)	10 (90.9)	0.626
Insufficient time following delivery	17 (65.4)	7 (43.8)	0.210
Increased workload on medical or midwifery staff	22 (71)	7 (38.9)	0.038
Lack of necessary equipment	22 (66.7)	10 (71.4)	1
Lack of necessary expertise	20 (69)	6 (42.9)	0.182
Encroachment of technology in to the birth process	11 (44)	8 (44.4)	1
Decreased patient contact time	10 (35.7)	5 (25)	0.535

^a^Comparing rate of change with negative responses, neutral responses removed from analysis

**Table 3:** Identification of barriers to implementing universal umbilical artery lactate analysis pre and post training (the influence of clinical role)

Barrier	Pre training		*p*-value	Post training		*p*-value
	Midwife *n* (%)	Doctor *n* (%)		Midwife *n* (%)	Doctor *n* (%)	
Expense	9 (69.2)	6 (100)	0.255	5 (100)	5 (83.3)	1
Insufficient time following delivery	13 (72.2)	4 (50)	0.382	7 (77.8)	0 (0)	0.003
Increased workload on medical or midwifery staff	15 (75)	7 (63.6)	0.683	5 (50)	2 (25)	0.367
Lack of necessary equipment	16 (80)	6 (46.2)	0.065	6 (85.7)	4 (57.1)	0.559
Lack of necessary expertise	14 (82.4)	6 (50)	0.106	6 (85.7)	0 (0)	0.005
Encroachment of technology in to the birth process	11 (73.3)	0 (0)	0.001	8 (88.9)	0 (0)	<0.001
Decreased patient contact time	10 (55.6)	0 (0)	0.004	5 (45.5)	0 (0)	0.038

Gender influenced only two of the 22 individual Likert items. Prior to training, females were more likely to identify decreased patient contact time as a barrier to implementation of UA lactate analysis (positive response 10(52.6 %) vs. 0(0 %), negative response 9(47.4 %) vs. 9(100 %), *p* = 0.010). Following training, females were more likely to identify lack of equipment as a barrier (positive response 9(90 %) vs. 1(25 %), negative response 1(10 %) vs. 3(75 %), *p* = 0.041). Respondents length of time providing intrapartum care influenced attitude prior to training towards encroachment on the birth process being a barrier to UA lactate analysis; a negative response (disagree or strongly disagree) was seen in 7(53.8 %) and 5(100 %) of those providing care for <1 year and 1–5 years respectively, but in 0(0 %) and 1 (33.3 %) of those providing care for 6–10 years and >10 years respectively (*p* = 0.049). This same relationship did not persist following training.

On multivariate analysis, we looked at the role of gender, age, clinical position, and length of time practicing on each barrier to implementation pre- and post-training using ordinal regression models. Prior to training, the predictive value of a negative log-log model was significant only for the outcome of decreased patient contact time, where being male made it more likely that one would disagree with this being a barrier (coeff. est. 1.13 95 % CI 0.004, 2.25, *p* = 0.049), and being a midwife made it significantly less likely that one would disagree with this being a barrier (coeff. est. -1.31 95 % CI -2.58, -0.5, *p* = 0.42). Following training, when adjusted for age, gender, and length of time practicing, midwives were less likely to disagree that time following delivery would be a barrier to implementation of lactate analysis (coeff. est. -2.31 95 % CI -4.45, -0.47, *p* = 0.01). A shorter time practicing was associated with lack of equipment being less likely to be considered a barrier post training.

Of those that responded to the item in the post training questionnaire, all (*n* = 20) strongly agreed or agreed that UA lactate analysis was beneficial to maternity care. Nineteen participants responded to the negative item (that UA lactate analysis had no place in maternity care), with most (*n* = 15) disagreeing, two remained neutral and two participants agreed. Finally, participants were offered free form sections in the post training questionnaire asking about their overall opinion regarding introduction of UA lactate analysis and what they saw as the major barrier to the success of this. Of the 18 overall opinion comments, 17 were positive that it was feasible and that it would “add to knowledge”, “improve our standards”, “show(s) the impact of decision making” and “help(s) midwives to manage deliveries well”. One respondent said “due to shortage, it is impossible” and four respondents raised concerns about the cost or potential disruptions in supply of equipment. There were 17 replies regarding potential barriers; 8 raised cost and / or resources as the greatest barrier. Two respondents commented on the need for team will and overcoming the barrier of making UA lactate analysis a part of routine practice.

## Discussion

Taking evidence beyond a research setting and implementing it into clinical practice potentially requires complex institutional and behavioural changes [27], and the attitudes of staff towards any implementation will inform and influence this process. We have assessed the attitudes of medical and midwifery/nursing staff in a South African district level hospital towards the use of umbilical artery lactate as a measure of intrapartum care. District hospitals comprise the bulk of South African hospitals (75 %) and deliver almost 41 % of the nearly one million annual births in South Africa, and overall have a perinatal mortality rate of 24.3:1000 [28]. Given the response rate and the characteristics of Zithulele Hospital, we have captured the attitudes of staff to the implementation of UA lactate in a setting that as closely as possible reflects usual care at this level in South Africa.

There were overwhelmingly positive attitudes towards the staff’s ability for CTG interpretation, despite the relatively high perinatal mortality rate in the unit. Additionally, the role of audit was seen positively yet respondents felt more could be done about adverse outcomes secondary to intrapartum asphyxia. Perhaps this goes some way to explaining the vastly positive attitudes towards the benefits of implementing UA lactate in this setting; there is widespread awareness of the rate of hypoxia (intrapartum asphyxia is second only to unexplained stillbirths as the cause of perinatal mortality in district hospitals in South Africa [28]) and consequently the responses may reflect a general willingness to embrace anything that may be seen as a potential solution to this. Certainly this explanation is supported by the free form comments in both the pre and post training surveys, with many respondents expressing excitement over a tool that may reduce mortality, as well as having a tool which may give some information as to why a baby is born in poor condition. While the use of UA lactate would be a small step in a complex pathway to reduce perinatal mortality, this attitude may mean that implementation outside of the research setting is more likely to be successful.

The ability to identify and overcome barriers is critical to the implementation process. We have showed the role that training can play in this process, particularly around the perceived barrier of increased workload on medical or midwifery staff. That training had a trend towards decreasing time and expertise being seen as barriers is encouraging; it may be possible to overcome these barriers to ensure successful implementation. Training in this setting had no impact on midwives perceiving encroachment of technology into the birth process and decreased patient contact time being potential barriers to implementation of UA lactate. White et al. found that midwives in their setting (tertiary and secondary hospitals in Western Australia, Australia) were also likely to perceive encroachment of technology into the birth process as a barrier to implementation [20]. Given the role midwives play in caring for women who remain physiologically normal, it seems likely that we would be unlikely to remove this as a barrier should implementation of UA lactate be universal (i.e. to be taken following births perceived as normal as well as those ones considered high risk where medical staff are already involved). The decreased patient contact time is certainly an issue that deserves much consideration in implementation in this setting: world bank data from 2013 reports South Africa having a ratio of nurses and midwives as 5.1 per 1000 people [29], which, while there is no clear definition of the ideal ratio, remains well below reported rates in high income countries [30]. It is possible that as time progresses, this would be less likely to be seen as a barrier as staff time spent on sampling shortened with experience.

The strength of this study lies in the participation of nearly all care-providers involved in intrapartum care in this setting. The attitudes to UA lactate implementation are drawn from an environment reflective of the most common setting for intrapartum care in South Africa. However, the single site setting of this study, and the small size of the hospital also contributes to the potential limitation and bias in this study; the trend towards positive answers may be a consequence of a Hawthorne effect, with staff more inclined to be positive if it would be perceived that dissenters would be easily identified amongst a small number of participants. Moreover, those that did not respond to the post-training questionnaire may have had negative responses to the questionnaire. The author (KG) who conducted the lectures and teaching was also known to and trusted by the staff, which may have added to their positive response. This study is also limited by the small numbers; the role of gender and age on barriers should be interpreted cautiously, given the significant majority of respondents were females under the age of 40 (although this may be reflective of the usual demographics of intrapartum care providers in South Africa). If wider use of UA lactate sampling is considered, then a multi-centre study to explore and address barriers to implementation may be of benefit to clinicians, researchers, and policy makers.

## Conclusion

The majority of participants providing intrapartum care in this setting are positive about the role of universal UA lactate analysis and the potential benefits it provides. Training concentrating on fetal physiology, CTG interpretation and the process of UA lactate analysis aids in overcoming some of the perceived barriers to implementation of universal UA lactate analysis. Other barriers to implementation identified in this study may help in the planning of an effective implementation process.

## Abbreviations

CTG, cardiotocograph; UA, umbilical artery
